# PbrMYB186 activation of PbrF3H increased flavonol biosynthesis and promoted pollen tube growth in *Pyrus*

**DOI:** 10.1186/s43897-024-00110-6

**Published:** 2024-08-20

**Authors:** Xueying Liu, Hao Zhang, Zhuqin Liu, Chao Tang, Shouzheng Lv, Ming Qian, Ningyi Zhang, Shaoling Zhang, Juyou Wu, Peng Wang

**Affiliations:** 1 Sanya Institute of Nanjing Agricultural University, State Key Laboratory of Crop Genetics & Germplasm Enhancement and Utilization, Jiangsu Key Laboratory for Horticultural Crop Breeding, College of Horticulture, Nanjing Agricultural University, Nanjing, Jiangsu 210095 China; 2 Ningbo Key Laboratory of Characteristic Horticultural Crops in Quality Adjustment and Resistance Breeding, Ningbo Academy of Agricultural Science, Ningbo, 315040 Zhejiang China; 3 Zhongshan Biological Breeding Laboratory, Nanjing, Jiangsu 210014 China

Pear (*Pyrus bretschneideri*) is one of the important economic fruit trees in the Rosaceae family (Wu et al. 2013). However, pear is a typical gametophytic self-incompatible species that requires artificial cross-pollination to obtain the pear fruits, leading to a high labor cost during production (Chen et al. 2018; Wu et al. 2023). Elucidating the molecular mechanisms underlying pollen tube growth is essential to ensure the successful fertilization and fruit bearing.

Flavonoids is an important group of plant secondary metabolites that regulate numerous physiological processes, including plant development, reproduction and antioxidation. Mutations altering the synthesis of flavonoids, including flavonols and anthocyanins, have been found to disrupt pollen development (Muhlemann et al. 2018; Schijlen et al. 2007). Flavonoids facilitate pollen development by decreasing the abundance of reactive oxygen species (ROS) (Lan et al. 2017). Flavonoids also regulate sexual reproduction in plants at normal and high temperatures by maintaining ROS homeostasis (Muhlemann et al. 2018). However, the specific function of flavonoids in pollen tube growth and the molecular mechanisms of flavonoid biosynthesis in pear pollen remain unclear.

The 2-oxoglutarate-dependent dioxygenase (2OGD) enzyme family serves as crucial components in various metabolic processes, particularly in flavonoid biosynthesis (Kawai et al. 2014). Flavonoids, recognized for their contributions to plant coloration and their multiple functions in UV protection, plant immunity, and fertility, are synthesized through enzymatic action, notably by flavanone 3-hydroxylase (F3H) (Tohge et al. 2017; Muhlemann et al. 2018). The expression of *F3H* and other genes within the flavonoids synthesis pathway can be regulated by MYB transcription factors (Premathilake et al. 2020). While previous studies have reported that flavonoids play critical roles in pollen germination, growth, and fertility (Muhlemann et al. 2018; Schijlen et al. 2007; Lan et al. 2017), the precise molecular mechanism by which the MYB-F3H module regulates flavonoid biosynthesis in pear pollen remains elusive.

To investigate the regulation mechanism of flavonoid biosynthesis in pear pollen tube, we conducted a genome wide analysis of *2OGD* family in pear. A total of 214 *2OGD* genes were identified in the pear genome (Table S1). Pear *2OGD* genes were classified into three subgroups (DOXA, DOXB and DOXC) based on phylogenetic and structural features. Further analysis within the DOXC subgroup revealed 11 subclasses, including AOP, DAO, GA2ox, GA20ox, GA3ox, BX6, NCS, FLS/ANS, F3H, ACO, and FNS/S3H/H6H (Fig. S1 and S2).

Further analysis of the evolutionary history of the *2OGD* family members in pear showed that most of the Ka/Ks of all *2OGD* gene pairs (except Pbr011717.1-Pbr011715.1) were found to be less than 1 (Table S2), indicating that the pear *2OGD* family has undergone a long period of purifying selection. In the evolutionary history of pear, two large-scale whole genome duplication (WGD) events have occurred (Wu et al. 2013), and the Ks values (0.0086–0.5771) of 88 homologous gene pairs (50.29%) of the *2OGD* family were distributed in the recent WGD (Ks ~ 0.15–0.3) event (Table S2), resulting in the expansion of pear *2OGD* family members.

Based on transcriptome data from various tissues of pear (Wang et al. 2023; Zhou et al. 2016), we observed that *PbrF3H* and *PbrFLS1* were highly expressed in pear pollen (Figs. 1A; S3). The expression pattern suggested that *PbrF3H* and *PbrFLS1* genes may be involved in the growth of pear pollen tubes.

**Fig. 1:**
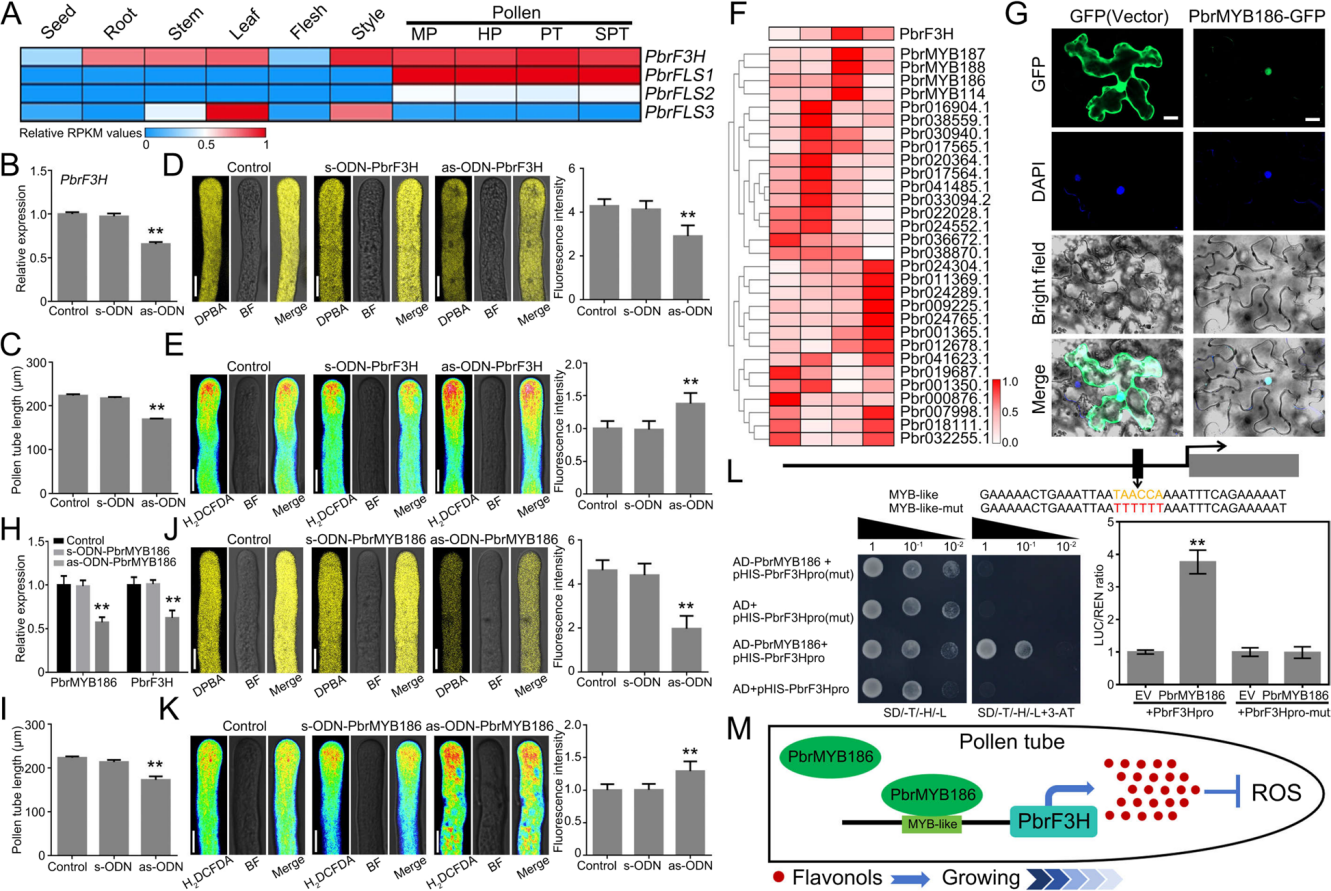
**A** Expression patterns of *F3H* and *FLS* genes in various tissues and pollen at different growth stages in pear. Highest expression level for each tissue was set to 1 as a standard for normalization. Red and blue represent high and low expression levels, respectively. MP (mature pollen), HP (hydrated pollen), PT (pollen tubes growing 6 h after hydration) and SPT (stopped growing pollen tubes) represent four stages of pear pollen growth. **B** *PbrF3H* expression decreased after as-ODN-PbrF3H treatment. **C** Statistics of pollen tube length after as-ODN-PbrF3H treatment. Significant differences (*p* < 0.01) by Student's *t*-test indicated as "**". **D** Knockdown of *PbrF3H* expression induces diminished pollen tube DPBA fluorescence intensity, bar = 10 μm. Significant differences (*p* < 0.01) by Student's *t*-test indicated as "**". **E** Knockdown of *PbrF3H* expression increases ROS levels in pollen tubes, bar = 10 μm. **F** Cluster analysis of *PbrF3H* and *MYB* genes in pear pollen at different growth stages. Red represents high expression level.** G** Subcellular localization analysis of the PbrMYB186, bar = 20 μm. **H** *PbrMYB186* and *PbrF3H* expression decreased after as-ODN-PbrMYB186 treatment. **I** Statistics of pollen tube length after as-ODN-PbrMYB186 treatment. Significant differences (*p* < 0.01) by Student's *t*-test indicated as "**". **J** Knockdown of *PbrMYB186* expression induces diminished pollen tube DPBA fluorescence intensity, bar = 10 μm. Significant differences (*p* < 0.01) by Student's *t*-test indicated as "**". **K** Knockdown of *PbrMYB186* expression increases ROS levels in pollen tubes, bar = 10 μm. **L** *PbrMYB186* binds to the *PbrF3H* promoter and activates its expression as demonstrated by yeast one-hybrid and dual-luciferase reporter assays. EV indicates empty vector. Significant differences (*p* < 0.01) by Student's *t*-test indicated as "**". **M** Model of PbrMYB186-PbF3H-flavonoid signaling pathway in pear pollen tubes. During the growth of pear pollen tubes, *PbrMYB186* directly binds to and activates the MYB-like element on the promoter of the *PbrF3H*. This activation promotes the expression of the *PbrF3H* gene regulating the production of flavonoids and ROS levels, and ultimately promoting pollen tube growth

To investigate the physiological functions of PbrF3H and PbrFLS, we performed subcellular localization assay, and found that *PbrF3H* was predominantly localized in the cytoplasm and nucleus, whereas *PbrFLS1, PbrFLS2* and *PbrFLS3* were mainly localized in the nucleus (Fig. S4). To investigate the function of the PbrF3H and PbrFLS1 in pollen tube growth, we used antisense oligonucleotide (as-ODN) methods to knock down their expression levels in pear pollen. Knockdown of *PbrF3H* expression in pollen tubes led to significant reductions in flavonol content and pollen tube length (Fig. 1B-D). Similarly, silencing *PbrFLS1* expression in pollen tubes resulted in reduced flavonol content and inhibited pollen tube growth (Fig. S5). Collectively, these findings indicated that the *PbrF3H* and *PbrFLS1* genes were essential for flavonoid biosynthesis and pollen tubes growth in pear.

Flavonoid biosynthesis is determined by structural genes, which in turn are closely related to *MYB* transcription factors. Using pear pollen transcriptome data (Zhou et al. 2016), the average FPKM values of *MYB* family members in pollen were clustered and analyzed, leading to the identification of four candidate transcription factors (*PbrMYB186*, *PbrMYB187*, *PbrMYB188* and *PbrMYB114*) potentially involved in regulating flavonoid synthesis with conserved MYB domains (Fig. 1F). Meanwhile, *PbrMYB186* and *PbrMYB187* showed similar expression pattern to *PbrF3H*, with all three genes were highly expressed in pollen tubes (Fig. S6A). Additionally, through dual-luciferase reporter (DLR) assay, PbrMYB186 and PbrMYB187 could transcriptionally activate PbrF3H, with the LUC/REN values of PbrMYB186 about fourfold higher than the control (Fig. S6B). Simultaneously, the expression of *PbrMYB186* was tenfold higher than *PbrMYB187* (Fig. S6C). Therefore, we hypothesized that *PbrMYB186* serves as the predominant MYB transcription factor regulating the *PbrF3H* gene.

PbrMYB186 contains typical R2 and R3 domains characteristic of the R2R3-MYB subfamily (Fig. S7), with nuclear localization (Fig. 1G). To investigate the function of PbrMYB186 in flavonols accumulation, we performed an as-ODN assay on PbrMYB186 in pollen. Knockdown of *PbrMYB186* expression significantly reduced the relative expression of *PbrF3H* and flavonol content in pollen tubes, and ultimately led to the inhibition of pollen tube growth (Fig. 1H-I). These findings suggested that PbrMYB186 may act as a positive regulator of flavonol synthesis by activating the expression of the *PbrF3H* gene.

To verify whether flavonoids affect pollen growth through the level of ROS, we treated pollen tubes with as-ODN-PbrF3H or as-ODN-PbrMYB186 and observed a notable increase in ROS levels using H_2_DCFDA staining (Figs. 1E; K). Additionally, mass spectrometry analysis of pollen tubes post-as-ODN-PbrMYB186 and as-ODN-PbrF3H treatments revealed alterations in flavonoid species distribution, notably decreasing levels of kaempferol and quercetin (Fig. S8A). The growth inhibition phenotype of pollen tubes was rescued by in vitro kaempferol supplementation to as-ODN-PbrMYB186 and as-ODN-PbrF3H-treated pollen medium (Fig. S8B). These findings underscore the indispensable role of flavonoids in pollen growth.

To tested whether *PbrF3H* was a direct target of PbrMYB186, we performed yeast one-hybrid assay and electrophoretic mobility shift assay (EMSA). The result indicated that PbrMYB186 bind to the *PbrF3H* promoter at conserved MYB binding site (TAACCA) (Fig. 1L). Subsequently, DLR analysis indicated that PbrMYB186 activated PbrF3H promoter four-fold compared with MYB-like elements mutant control (PbrF3H-mut) (Fig. 1L). EMSA confirmed that PbrMYB186 recognize and specifically bind to the *PbrF3H* promoter MYB-like element (Fig. S9). These results suggested that PbrMYB186 was a transcriptional activator of the *PbrF3H*.

In summary, our findings revealed a molecular mechanism of PbrMYB186-PbrF3H-flavonoid signaling pathway in pear pollen tubes (Fig. 1M). During the growth of pear pollen tubes, PbrMYB186 directly bind to and activates the MYB-like element in the promoter of the *PbrF3H*. This activation promoted the expression of the *PbrF3H* gene regulating the production of flavonoids and ROS, and ultimately promoted pollen tube growth. Thus, this study elucidated the function of the PbrMYB186-PbrF3H-flavonol signaling pathway in pear pollen tubes, which contributes to the understanding of the regulatory network of flavonoids on pollen tubes growth.

### Supplementary Information

Supplementary Material 1.

Supplementary Material 2.

## Data Availability

The data and materials will be available upon reasonable request.
