# Supplementary material for: PbrMYB186 activation of PbrF3H increased flavonol biosynthesis and promoted pollen tube growth in Pyrus
Source: Mol Hortic. 2024 Aug 20;4:30. doi: 10.1186/s43897-024-00110-6 (PMC11334369; doi:10.1186/s43897-024-00110-6)
Supplement: Supplementary file 1 — Supplementary Material 1. [file 43897_2024_110_MOESM1_ESM.docx]

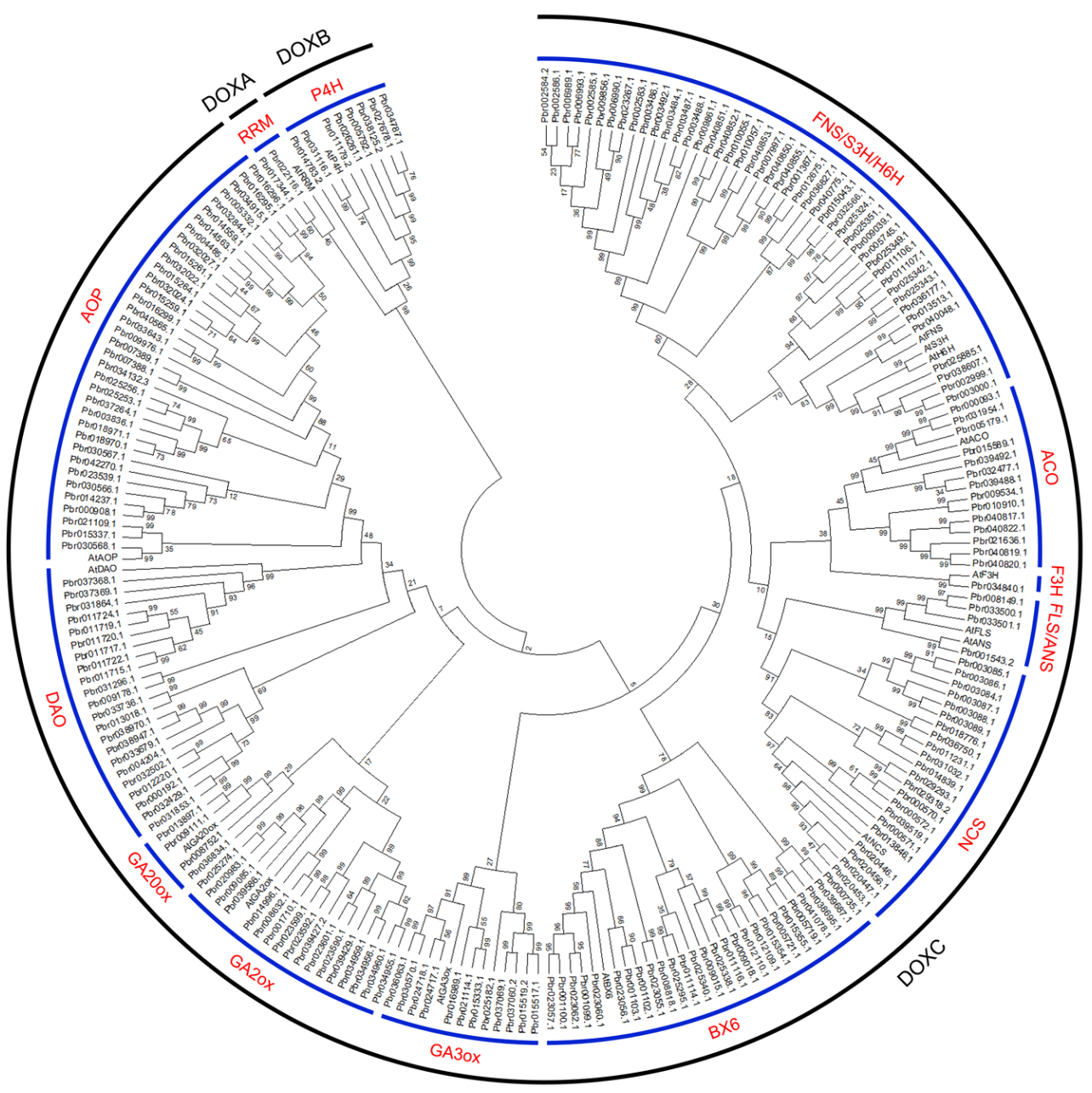


**Supplementary Fig. S1.** Phylogenetic tree of *2OGD* superfamily genes in pear and Arabidopsis. MEGA 7.0 software was employed to build phylogenetic tree, and take 1000 internal branches. The black letters represent three large subfamilies, and the red letters represent 13 small subfamilies.


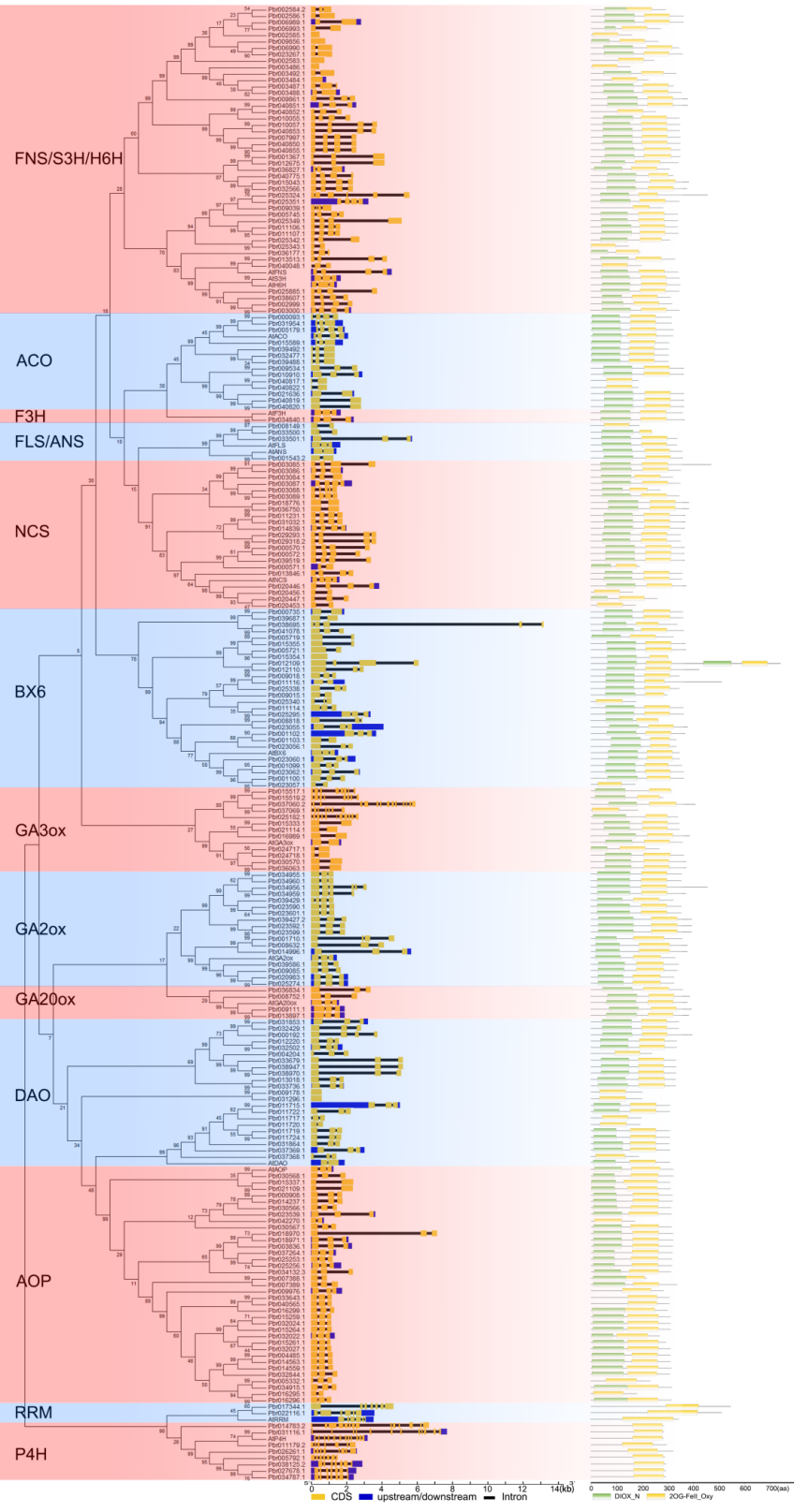


**Supplementary Fig. S2.** Analysis of the gene structure and protein conservative domains of the *2OGD* superfamily in pear and Arabidopsis. Phylogenetic tree was constructed by MEGA 7.0. Exons and introns were represented by yellow rectangles and black bold lines. The non-coding region was represented by a green rectangle. Domain DIOX_ N and 2OG-FeII_Oxy was represented by green and yellow rectangles, and the full-length sequences of the protein was represented by thin black lines.


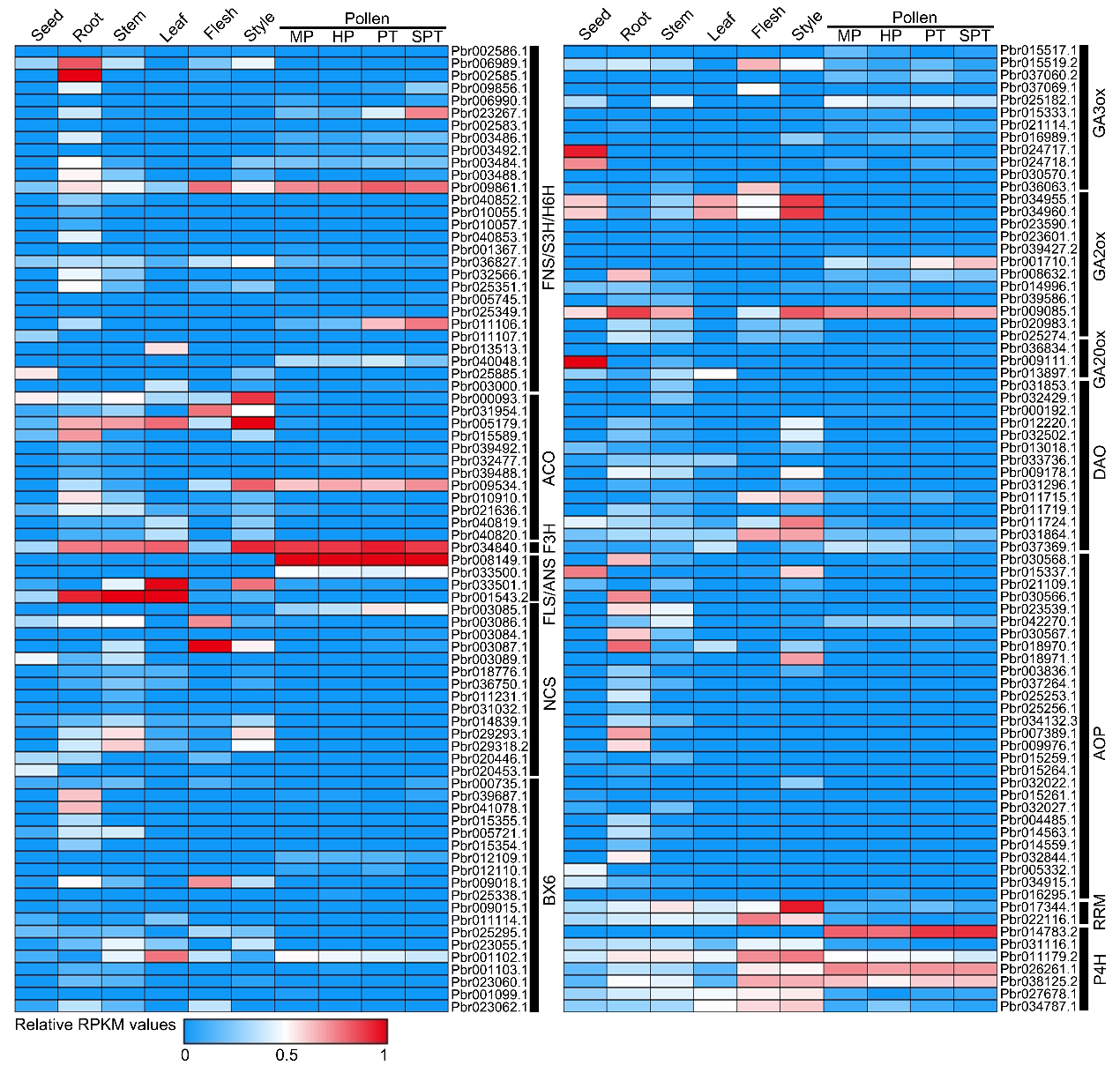


**Supplementary Fig. S3.** Expression patterns of *2OGD* genes in various tissues and pollen at different growth stages in pear. Highest expression level for each tissue was set to 1 as a standard for normalization. Red and blue represent high and low expression levels, respectively. MP (mature pollen grains), HP (hydrated pollen), PT (pollen tubes growing 6 h after hydration) and SPT (stopped growing pollen tubes) represent four stages of pear pollen growth.


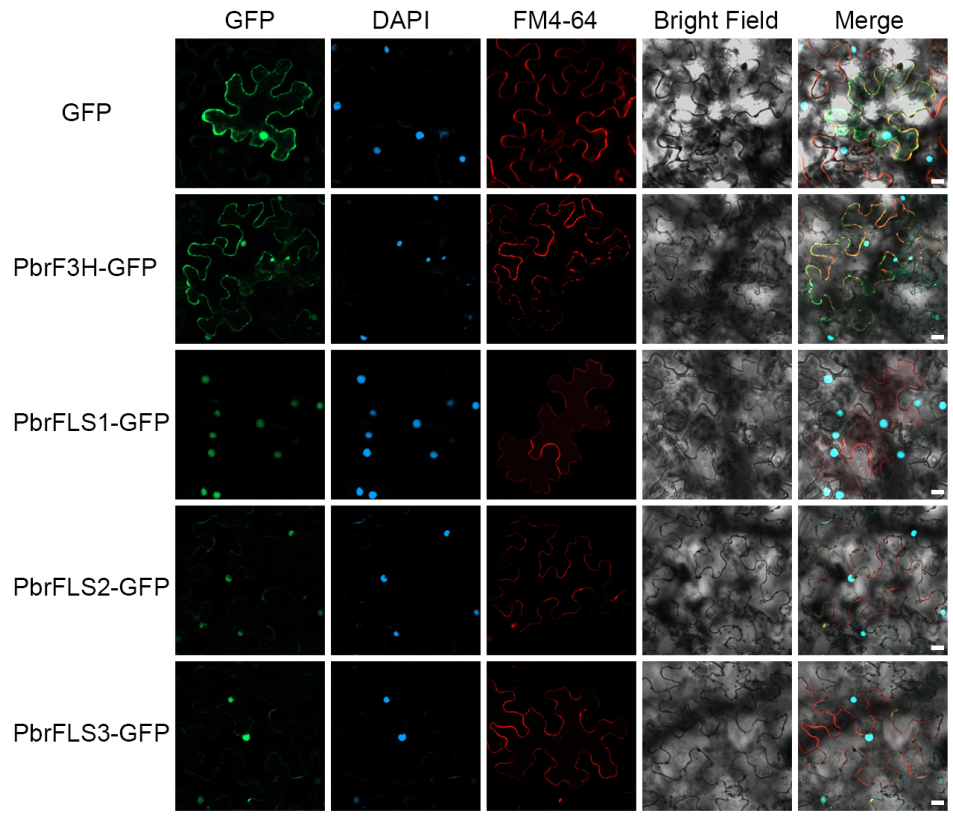


**Supplementary Fig. S4.** Subcellular localization of PbrF3H and PbrFLS1-3 proteins. DAPI and FM4-64 were used as nuclear and plasma membrane markers, scale bar = 20 μm.


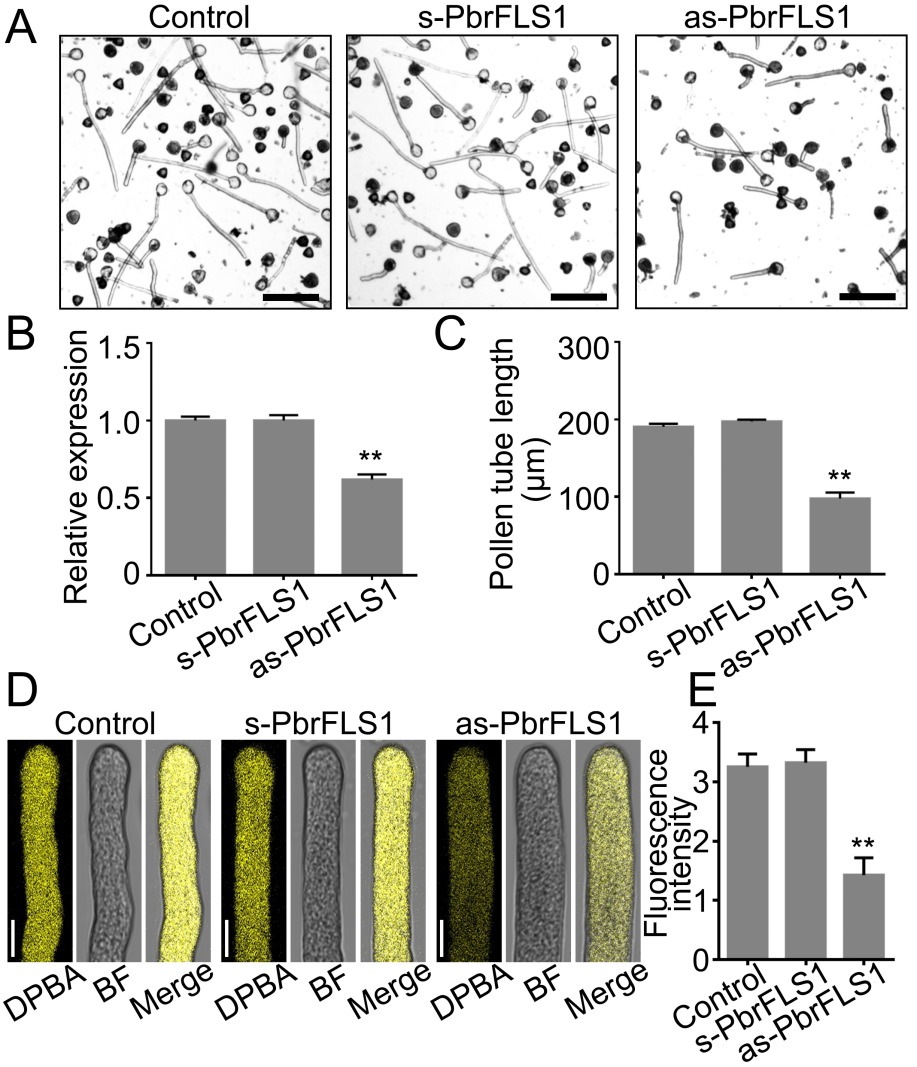


**Supplementary Fig. S5.** Knockdown of PbrFLS1 gene expression inhibits pollen tube growth in pear. **A** The as-ODN-PbrFLS1 treatment inhibits pollen tube growth, bar = 100 μm. **B** PbrFLS1 expression decreased after ODN treatment. **C** Statistics of pollen tube length. **D** Knockdown of PbrFLS1 expression induces diminished pollen tube DPBA fluorescence intensity, bar = 10 μm. **E** Quantification of the DPBA fluorescence intensity. Significant differences (*p* < 0.01) by Student's *t*-test indicated as "**".


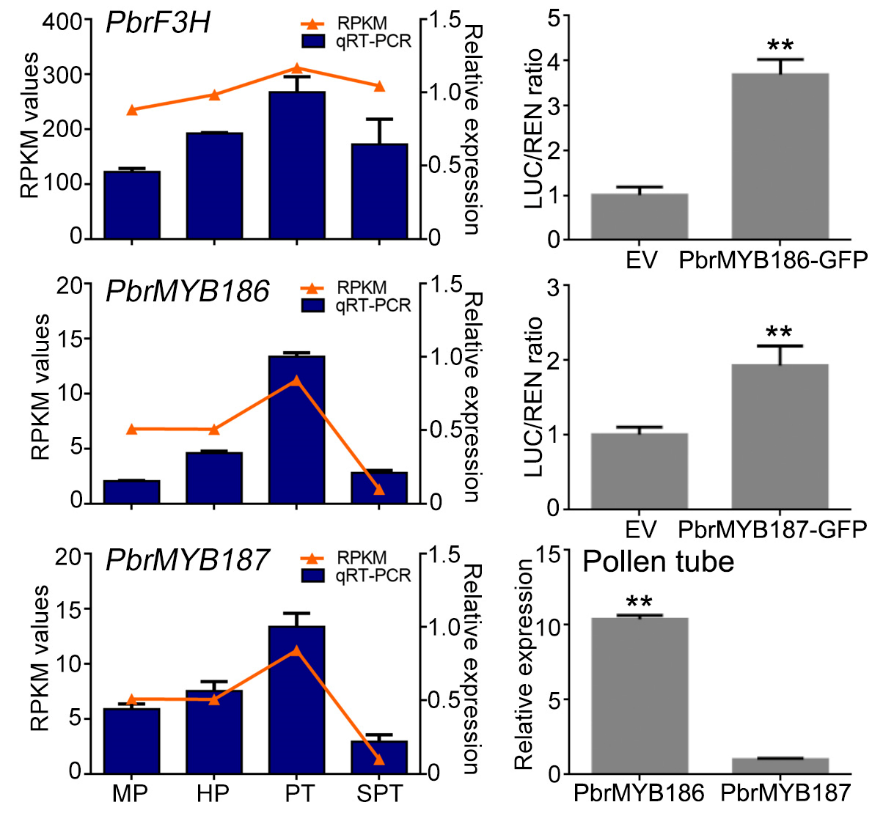


**Supplementary Fig. S6.** Expression levels and functional validation of genes from PbrMYB186 and PbrMYB187 during pollen growth. **A** Expression analysis of *PbrF3H*, *PbrMYB186* and *PbrMYB187* during pollen growth. **B** PbrMYB186 and PbrMYB187 both activate PbrF3H promoter activity. EV indicates empty vector. **C** Relative expression of *PbrMYB186* and *PbrMYB187* in pollen tube growth. Significant differences (*p* < 0.01) by Student's *t*-test indicated as "**".


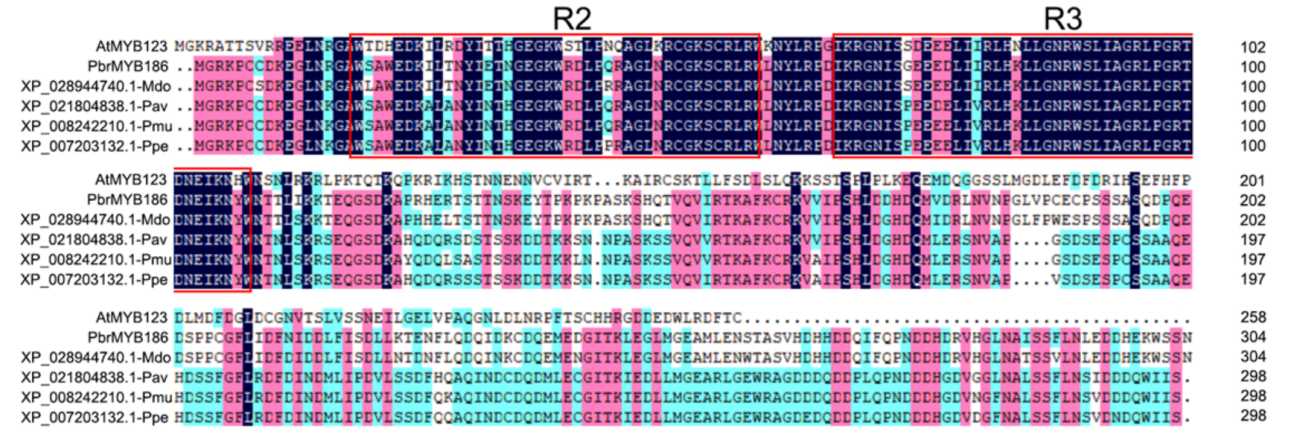


**Supplementary Fig. S7.** Amino acid multiple sequence analysis of the PbrMYB186 proteins, red boxes represent the MYB R2 and R3 structural domains in the PbrMYB186 coding sequence.


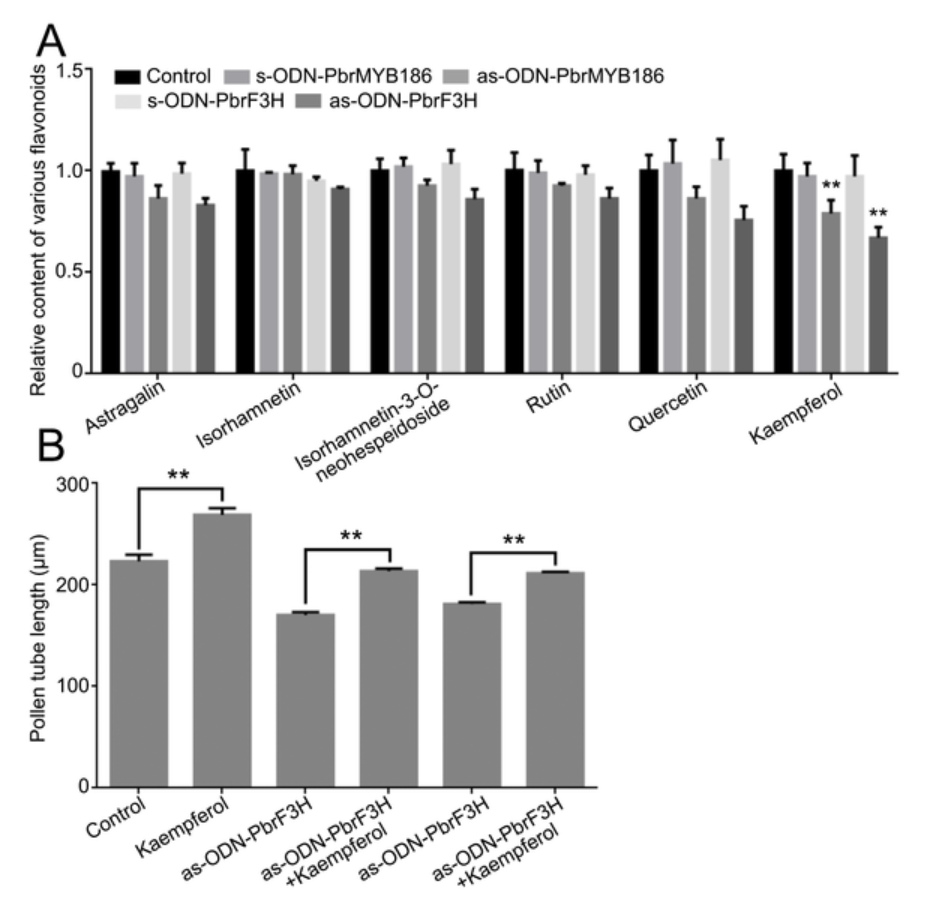


**Supplementary Fig. S8.** Exogenous kaempferol rescues the phenotype of pollen tube growth after as-ODN-PbrMYB186 and as-ODN-PbrF3H treatments. **A** Effect of knocking down the expression of PbrMYB186 and PbrF3H on the content of different flavonoids in pear pollen. **B** Exogenous kaempferol was able to backfill the phenotype of inhibited pollen tube growth after as-ODN-PbrMYB186 and as-ODN-PbrF3H treatments. Significant differences (*p* < 0.01) by Student's *t*-test indicated as "**".


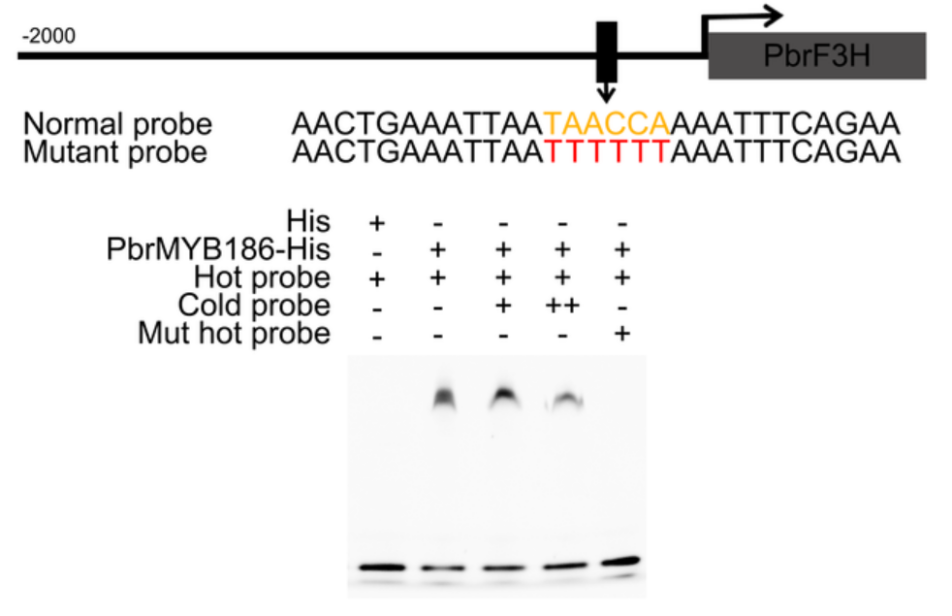


**Supplementary Fig. S9.** Electrophoretic mobility shift assay (EMSA) confirmed that PbrMYB186 binds to the TAACCA motif within the PbrF3H promoter. Symbols + or - indicate the absence or presence of recombinant PbrMYB186-His protein, biotin-labeled probe, biotin-labeled mutant, or cold probe, respectively. Cold probe concentrations were 10-fold (+) and 100-fold (++) that of the labeled probe.

**Materials and methods**

1. **Plant materials and quantitative real-time PCR (qRT-PCR)**

Pear expression profiles of different tissues were obtained from the Pear Expression Database (<http://www.peardb.org.cn/>) (Wang et al. 2023), and transcriptomic data of different pear pollen growth stages from published paper (Zhou et al. 2016). Mature pollens were collected from pear trees (*Pyrus bretschneideri*) in the experimental field of Nanjing Agricultural University, China. Pollen medium consists of 5 mM 2-(N-morpholino) ethanesulfonic acid hydrate, 1.5 mM H_3_BO_3_, 0.5 mM Ca(NO_3_)_2_ and 450 mM sucrose at pH = 6.2 (adjusted with Tris). For pollen growth experiments as previously described (Zhou et al. 2016), we collected pollen samples at 0 h (MP, mature pollen grains), 0.67 h (HP, hydrated pollen), 6 h (PT, pollen tubes growing 6 h after hydration), and 15 h (SPT, stopped growing pollen tubes), respectively. Expression analysis experiments were performed on pollen at different growth stages by qRT-PCR. Extraction of total RNAs with RNA isolation kits (Vazyme, China). Subsequently, the samples were reverse transcribed according to the manual of the HiScript^®^ III RT SuperMix for qPCR kits (Vazyme, China). Gene-specific primers for *PbrFLS1*, *PbrF3H* and *PbrMYB186* (Table S3) were confirmed on the pear genome website. The qRT-PCR was performed on a LC480 II (Roche, Germany) and SYBR Green qPCR Master Mix (Vazyme, China), using *PbrUBQ* and *PbrEF1α* genes as internal control. Expression data were calculated using the 2^-ΔΔCt^ method.

1. **Identification and bioinformatics analysis of *2OGD* genes in pear**

To identify *2OGDs* in pears, a conserved structural domain (Pfam: PF03171) was performed in a Hidden Markov Model to search for candidate *2OGDs* with E-values < 1e^-10^. Finally, 214 *2OGDs* were characterized by the PFAM (http://pfam.xfam.org/) and SMART (http://smart.embl-heidelberg.de/) websites to search for candidate genes with the 2OG-FeII_Oxy functional domain.

Phylogenetic trees of *2OGDs* from pear and Arabidopsis were constructed using the neighbor-joining method with MEGA 7.0 and reliability was tested by bootstrap method with 1,000 replications. In addition, GSDS 2.0 (http://gsds.gao-lab.org/) and PFAM were used to recognize the intron/exon structure and conserved domain of *2OGDs*, respectively (Hu et al. 2015).

Collinearity relationships analysis using the method developed by PGGD (Lee et al. 2013). Different duplication patterns of *2OGDs* were identified using MCScanX and data were visualized with Circos software (Krzywinski et al. 2009; Wang et al. 2012). The non-synonymous (Ka) and synonymous (Ks) values were calculated by KaKs_Calculator 2.0 (Qiao et al. 2019).

1. **Gene cloning and subcellular localization**

These genes *PbrF3H*, *PbrFLS1-3* and *PbrMYB186* were obtained through PCR amplification of pollen cDNA using high-fidelity phusion (Vazyme, China). The amplified fragments were then inserted into the pCAMBIA1300-35S: GFP vector between the Xba I and BamH I restriction sites. Through gene cloning in pear, it was found that there was an intron insertion in the annotation of the *Pbr041523.1* gene, which resulted in its misnaming as PbrMYB3R-10 (Li et al. 2016). Pbr041523.1 belongs to the R2R3 MYB family and was renamed PbrMYB186. Plant expression vectors (PbrF3H-GFP, PbrFLS1-3-GFP and PbrMYB186-GFP) were constructed by enzymatic digestion and ligation, and then the vectors were transformed into *Agrobacterium* GV3101 (Weidi, China). Successfully transformed *Agrobacterium* was incubated at 28°C for 24 h, washed and resuspended in liquid medium consisted of 10 mM MES (pH = 5.6), 10 mM MgCl_2_ and 100 µM acetosyringone (Aldrich, USA). Resuspended *Agrobacterium* was incubated for another 4 h at 25°C and 50 rpm and injected into tobacco leaves. After 2 days of infiltration, DAPI and FM4-64 was transfected and tobacco leaves were visualized by an LSM800 confocal microscope (Zeiss, Germany).

1. **The antisense oligodeoxynucleotide (as-ODN) assays**

The as-ODN experimental method as previously described (Chen et al. 2018). ODN sequences of *PbrFLS1*, *PbrF3H* and *PbrMYB186* (Table S3) were designed based on the RNAfold (<https://rna.tbi.univie.ac.at/cgi-bin/RNAWebSuite/RNAfold.cgi>). ODN primers were mixed with Lipofectamine 2000 (Thermo Fisher Scientific, USA) and incubated for 15 min, then added to pre-cultured pollen medium for 45 min to continue the incubation 2 h, with a final concentration of 30 µM ODN primer. The samples from various treatments were examined using a Nikon Eclipse E100 microscope (Tokyo, Japan), and the lengths of at least 50 pollen tubes were measured using Image ProPlus 6.0 software (Media Cybernetics, USA).

1. **Diphenylboric acid 2-amino ethyl ester (DPBA) staining for detection of flavonols in pollen tube**

For DPBA staining follow the previous description with minor modifications (Muhlemann et al. 2018). To the ODN-treated pollen tube samples, a final concentration of 20 μM DPBA was added and incubated for 30 minutes. The samples were then washed three times with pollen medium and observed using an LSM800 confocal microscope, and relative average DPBA signal intensity in pollen tubes were calculated with a Zeiss tool.

1. **Yeast one-hybrid (Y1H) assay**

Y1H assays were carried out in accordance with the Matchmaker^®^ Gold yeast system manual. The promoter fragment of PbrF3H was inserted into the pHIS2 vector and PbrMYB186 CDS was inserted into the pGADT7 vector. Inhibition of promoter self-activation using 3-amino-1,2,4-triazole (3-AT). The Y187 yeast cells carrying recombinant plasmids PbrF3H-pHIS2 and PbrMYB186-pGADT7 were cultured on selective medium lacking Leu, Trp and His (SD/-L-T-H) to detect interactions, and the pGADT7 empty vector as a control. To test whether PbrMYB186 binds MYB-like element (TAACCA) in the PbrF3H promoter, MYB-like element in the PbrF3H promoter was mutated using the Rapid Mutation System kit (Vazyme, China). Primers used were listed in Table S3.

1. **Dual-luciferase reporter (DLR) assay**

For DLR assays, the promoter fragment of *PbrF3H* was cloned into the pGreenII 0800-LUC reporter vector and *PbrMYB186* CDS was inserted into the pCAMBIA1300-35S: GFP effector vector. Subsequently, *Agrobacterium* (GV3101) containing PbrF3H-pGreenII 0800-LUC and PbrMYB186-GFP was co-infiltrated into tobacco leaves. The SpectraMax iD5 microplate reader (Molecular Devices, USA) was used to measure the LUC/REN activity ratio.

1. **Electrophoretic mobility shift assay (EMSA)**

The EMSA were performed using a Light Shift Chemiluminescence EMSA kit (Beyotime). PbrMYB186 CDS was inserted into the pCold-TF vector with a HIS tag. PbrMYB186-His protein was mixed with biotin-labeled MYB-like probes and incubated at 25℃ for 30 min. acrylamide gel electrophoresis was performed to separate the free and bound probes. The probe sequences are listed in Table S3.

1. **ROS staining**

To detect the changes of ROS in pollen tubes, H_2_DCFDA (20 μM) was used to stain the pollen tubes at 25℃ for 20 min, and then the samples were washed three times with liquid medium, and ROS in the pollen tubes were observed using the LSM800, and fluorescence intensity of the pollen tube tips was calculated and normalized using the Zeiss tool.

1. **Statistical analysis**

Data were statistically analyzed using GraphPad Prism 6.01. Two-group comparisons were analyzed using Student's *t*-test.

1. **Gene & Accession Numbers**

Genes and Sequence data used in this article can be retrieved from Pear Genome Project (http://peargenome.njau.edu.cn/) under the following accession numbers in GenBank: PbrF3H (AGZ15308.1) and PbrMYB186 (XP_009361684.1).

**References:**

Chen J, Wang P, de Graaf BHJ, Zhang H, Jiao H, Tang C, et al. Phosphatidic acid counteracts S-RNase signaling in pollen by stabilizing the actin cytoskeleton. Plant Cell. 2018;30(5):1023-1039. https://doi:10.1105/tpc.18.00021.

Hu B, Jin J, Guo AY, Zhang H, Luo J, Gao G. GSDS 2.0: an upgraded gene feature visualization server. Bioinformatics. 2015;31 (8):1296-1297. https://doi:10.1093/bioinformatics/btu817.

Krzywinski M, Schein J, Birol I, Connors J, Gascoyne R, Horsman D, et al. Circos: an information aesthetic for comparative genomics. Genome Res. 2009,19(9):1639-1645. https://doi:10.1101/gr.092759.109.

Lee TH, Tang H, Wang X, Paterson AH. PGDD: a database of gene and genome duplication in plants. Nucleic Acids Res. 2013;41:D1152-1158. <https://doi:10.1093/nar/gks1104>.

Li X, Xue C, Li J, Qiao X, Li L, Yu L, et al. Genome-wide identification, evolution and functional divergence of MYB transcription factors in Chinese white pear (*Pyrus bretschneideri*). Plant Cell Physiol. 2016;57(4) :824-847. https://doi:10.1093/pcp/pcw029.

Muhlemann J, Younts T, Muday G. Flavonols control pollen tube growth and integrity by regulating ROS homeostasis during high-temperature stress. P Natl Acad Sci Usa. 2018;115(47):E11188-E11197. https://doi:doi:10.1073/pnas.1811492115.

Qiao X, Li Q, Yin H, Qi K, Li L, Wang R, et al. Gene duplication and evolution in recurring polyploidization-diploidization cycles in plants. Genome Biol. 2019;20(1):38. https://doi:10.1186/s13059-019-1650-2.

Wang P, Wu X, Shi Z, Tao S, Liu Z, Qi K, et al. A large-scale proteogenomic atlas of pear. Mol Plant. 2023;16(3):599-615. https://doi:10.1016/j.molp.2023.01.011.

Wang Y, Tang H, Debarry J, Tan X, Li J, Wang X, et al. MCScanX: a toolkit for detection and evolutionary analysis of gene synteny and collinearity. Nucleic Acids Res. 2012;40(7):e49. https://doi:10.1093/nar/gkr1293.

Zhou H, Yin H, Chen J, Liu X, Gao Y, Wu J, et al. Gene-expression profile of developing pollen tube of *Pyrus bretschneideri*. Gene Expr Patterns. 2016;20(1):11-21. https://doi:10.1016/j.gep.2015.10.004.
